# Repurposing drugs during the COVID-19 pandemic and beyond

**DOI:** 10.4155/ppa-2020-0031

**Published:** 2021-01-15

**Authors:** Srividya Ravi, Sammita Jadhav, Anuradha Vaidya, Ravindra Ghooi

**Affiliations:** ^1^Symbiosis School of Biological Sciences (SSBS) (formerly Symbiosis School of Biomedical Sciences), Symbiosis International (Deemed University), Gram-Lavale, Taluka-Mulshi Dist, Pune 412115, India; ^2^Symbiosis Institute of Health Sciences, Symbiosis International (Deemed University), Gram-Lavale, Taluka-Mulshi Dist, Pune 412115, India

**Keywords:** pandemic, patent claims, pharmaceutical industry, repurposing

## Overview

In December 2019, physicians in the Chinese city of Wuhan were treating patients infected by a novel virus, now known as SARS-CoV-2. This virus caused a new influenza-like disease termed COVID-19. By September 2020, globally more than 27 million people were infected and almost 0.9 million were dead [1]. There was neither an immediate availability of a medical cure to treat the infected nor a vaccine to prevent the spread of the disease. Most countries announced lockdowns severely impacting industrial activity and hence, the economy. Observing the rapid spread of the virus and the attendant mortality, in March 2020 the WHO declared COVID-19 a pandemic [2].

The search for an effective therapeutic agent to treat COVID-19 began as early as December 2019. The Wuhan University filed a second medical use patent for Remdesivir, an antiviral drug that had been patented by Gilead Sciences, USA in several countries. Remdesivir had been approved as an orphan drug by the US FDA to treat Ebola, which Gilead sought to rescind in March 2020 [3]. The efficacy of Remdesivir to treat COVID-19 had not been established conclusively till August 2020, though the drug has been granted an emergency use authorization by regulatory authorities of some countries. Another antiviral drug that has been evaluated against the SARS-CoV-2 virus is Favipiravir [4]. It is established to be effective in mildly symptomatic patients when administered early in the infection. This drug has also been issued an emergency use authorization for the treatment of COVID-19 by the regulatory authorities of some countries.

Currently, the pharmaceutical industry is pursuing the development of a vaccine to prevent the infection. The time required for a new drug to be discovered is about 10–12 years, while the time frame for the development of a new vaccine is even longer. However, an immediate remedy was needed to control the pandemic and to treat patients with severe symptoms, many times also suffering from comorbidities. Therefore, the pharmaceutical industry chose to repurpose existing drugs rather than searching for a new one. Earlier between February and May 2020 there were around 30 candidate drugs that were being repurposed for treating COVID-19 targeting the entry of the virus into human cells, its method of replication or the various effects it causes in the body [5]. To date, no candidate has exhibited the potential to treat the infection. In August 2020, few more new candidates had been identified for repurposing to treat COVID-19 [6]. Recent literature lists more than 100 small molecules and several other drugs, biological and vaccines being repurposed for treating COVID-19 and undergoing clinical trials [7].

The route of repurposing is advantageous over the traditional route of drug discovery in terms of cost and time [8]. Repurposing is estimated to provide a drug at 60% of the cost incurred for a new drug discovery program, taking 3–4 years, which translates into about a third of the time taken for the usual route. The risk in traditional drug discovery is also reduced substantially since the repurposed drug has already been established to be safe. The drug only needs to satisfy the criterion of efficacy for the new indication through phased clinical trials. Therefore, it can be a viable route to provide expressly needed remedies at such times as the current pandemic, and also for treating diseases for which available therapies are unaffordable to patients in developing countries.

However, the repurposing route still does not seem to attract the pharmaceutical industry as well as it could have been, due to commercial reasons.

## Reasons for repurposing not being attractive

New chemical or biological molecules are eligible for the strong protection that a product or composition of matter patent offers. Further, pharmaceutical innovator organizations build a portfolio around the new drug with patents protecting the process of synthesis, various conventional dosage forms, novel drug delivery systems, pure forms, enantiomers, isomers, crystalline forms, improved dissolution profiles and binding efficiencies to name a few. Such first use product patent portfolio is usually difficult to challenge and is well protected from potential infringement. The revenue accrued by virtue of this robust patent protection is necessary to ensure the sustainability of ongoing research and development programs [9].

Repurposed drugs are eligible for patent protection through use claims or method of treatment claims. The European Patent Organization (EPO) provides protection for second medical use by way of a Swiss claim which reads as ‘Use of substance X to manufacture a medicament to treat a condition Y’. The Patent Acts of the USA and Australia allow protection for second medical use through method claims for the treatment or prophylaxis of a disease. However, the patent laws of many countries including India, Indonesia and Argentina do not allow patent protection for repurposed drugs. Also, aligned with the Trade-Related Intellectual Property Rights (TRIPs), the patent laws of many countries do not provide patent protection for methods of treatment, diagnosis or prophylaxis [10,11]. The aforementioned method and use patents provide a limited and weak scope of protection, are easy to work around and difficult to enforce [12].

The drug regulations in the US provide market exclusivity of 3 years for the second indication. Europe provides an additional 1 year over the existing 8 years data exclusivity if the new use is invented within those 8 years. However, many countries including India do not have such provisions for repurposed drugs [13–15].

The case of Pregabalin judged by the Supreme Court of the UK is an excellent example of poor enforcement of second medical use patents. The verdict also provides adequate evidence in the matter of the challenges faced to detect infringement by the various stakeholders, namely the manufacturer, physician, pharmacist and patient. The Court concluded that none of the relevant stakeholders can be infringers, further emphasizing the challenges in the enforcement of such claims. One more significant conclusion drawn by the court was that when a second medical use is proposed, medicaments may need to be prescribed by their brand names, and not by their generic or international nonproprietary names [16]. On the other hand, to increase the affordability of medicines and address public health issues, the Indian Medical Council, the nodal agency to regulate medical practice in India requires that the medical practitioners prescribe medicaments by their generic names, clearly avoiding branding [17]. Thus, in such markets, especially where literacy levels are very poor, infringement (and its detection) of the rights of both the innovator and that of the proposer of the second medical use, remain unresolved issues.

Pharmaceutical innovator organizations are known to pursue research and seek regulatory approvals only for those candidates which are strongly protected through patents [12]. A survey of drugs approved in India between 1991 and 2011 showed that only 5% of the drugs approved were for second indication [18]. In conclusion, a combination of nonexisting or poor intellectual property rights protection, completely absent or short tenure of market/data exclusivity and ineffective mechanisms to detect infringement contribute to making repurposing a commercially unattractive pursuit.

### Proposals for repurposed drugs

Both pharmaceutical and legal researchers have attempted to overcome the issues faced in repurposing drugs and suggested different approaches to resolve the various challenges. One proposal is a compensation mechanism through complex calculations for the investments carried out to conduct clinical trials for a second medical use [19]. Another proposal relates to the process of discovering new drugs. It is recommended that pharmaceutical organizations should consider exploratory research for second medical use during the clinical trials for the first use itself, resulting in the saving of resources [20]. But there is no evidence of the practical implementation of either proposal. The provision of government funding or public-private partnership schemes has also been proposed by some researchers. Another critical initiative has been the sharing of molecular libraries between corporates and research institutions to test molecules for a possible second medical use. Such practices are being observed in a few instances, particularly in the US.

### Intellectual property rights related initiatives undertaken during the pandemic

In April 2020, Prof Carlos Correa, Director – South Center had appealed to the Director-General of WHO, Secretary-General – United Nations and to the United Nations High Commissioner for Human Rights that “Any commercial interest supported by the possession of intellectual property rights on those technologies must not take precedence over saving lives and upholding human rights to support developing and other countries, as they may need, to make use of Article 73(b) of the TRIPS Agreement to suspend the enforcement of any intellectual property right (including patents, designs and trade secrets) that may pose an obstacle to the procurement or local manufacturing of the products and devices necessary to protect their populations [21]”.

In October 2020, the Governments of India and South Africa submitted a communication to the World Trade Organization (WTO) titled “Waiver from certain provisions of the TRIPS agreement for the prevention, containment and treatment of COVID-19”. They sought to bring together the WTO members to ensure that the various forms of IP do not cause barriers for affordable access to medicines, vaccines, diagnostics, research, development, manufacturing and supply of products needed during this pandemic. This was supported by many countries and more than 300 organizations globally, but was objected to by countries such as the USA, UK and many other European countries [22]. The first meeting of the WTO members did not result in a consensus on the issue and the next session of the WTO is scheduled around mid-December.

However, there have been no proposals to amend the patentability criteria or patentable subject matter by way of any international agreements or national statutes.

## Future perspective

To incentivize pharmaceutical companies to vigorously pursue repurposing, immediately implementable remedies as well as various long-term measures should be undertaken.

The issue of infringement of second use patents can be prioritized by proposing amendments to labelling rules in national drug laws. A system to identify products approved for second medical use by overt or covert text, visual or coded symbols can be effected to protect the rights of the innovator as well as that of the proposer of the second medical use. This feature will enable detection of infringement independent of whether the products are marketed under a brand name or as a generic and in the same dose or dosage form.

On a mid-term basis, research and development-based pharmaceutical organizations should open their molecule libraries to researchers who want to study them for a possible second medical use. An international repository for new molecules should be actively considered and a deposition process facilitated for obtaining patent protection for new chemical entities. This will ease the availability of molecules for further research.

Eventually, over a period of time, a review of international treaties and harmonization of patentability criteria to provide a mechanism of protection for such discoveries can be considered. This is very relevant considering that even vaccines and traditional medicines are being repurposed, examples being the BCG vaccine and plants such as neem and ashwagandha for providing immunity against the COVID-19 [5].

Another option that should be debated at an international level is the current model of the drug discovery. With continuous advances made in the field of data sciences, computing, artificial intelligence, testing instrumentation and synthetic processes, such technologies should be actively applied to enhance the efficiency of the process of drug discovery.

In conclusion, a multi-pronged approach is required to reap the immense benefits of the repurposing route of drug discovery. This will create an alternate course of drug discovery, leading to affordable access to medicines and immense benefits to patients.
